# 2-[2-(3-Chloro­phen­yl)hydrazinyl­idene]-1,3-diphenyl­propane-1,3-dione

**DOI:** 10.1107/S1600536811017909

**Published:** 2011-05-20

**Authors:** Carlos Bustos, Luis Alvarez-Thon, Juan-Guillermo Cárcamo, Andrés Ibañez, Christian Sánchez

**Affiliations:** a Instituto de Ciencias Químicas, Universidad Austral de Chile, Avda. Los Robles s/n, Campus Isla Teja, Casilla 567, Valdivia, Chile; b Departamento de Ciencias Físicas, Universidad Andres Bello, Avda. República 220, Santiago de Chile, Chile; c Instituto de Ciencias Moleculares y Microbiología, Universidad Austral de Chile, Avda. Los Robles s/n, Campus Isla Teja, Casilla 567, Valdivia, Chile; d Laboratorio de Cristalografía, Difracción de Rayos-X, Departamento de Física, Facultad de Ciencias Físicas y Matemáticas, Universidad de Chile, Av. Blanco Encalada 2008, Santiago, Chile

## Abstract

The mol­ecular structure of the title compound, C_21_H_15_ClN_2_O_2_, features one strong intra­molecular N—H⋯O resonance-assisted hydrogen bond (RAHB). In the crystal, mol­ecules form inversion-related dimers *via* pairs of weak inter­molecular N—H⋯O contacts. These dimers are further stabilized *via* three weak C—H⋯O contacts, developing the three-dimensional structure.

## Related literature

For resonance-assisted hydrogen bonds, see: Bertolasi *et al.* (1993 ▶, 1994*a* ▶,*b* ▶); Inabe (1991 ▶); Krygowski *et al.* (1997 ▶); Olivieri *et al.* (1989 ▶). For details of the synthesis, see: Bustos *et al.* (2007 ▶, 2009 ▶); Yao (1964 ▶).
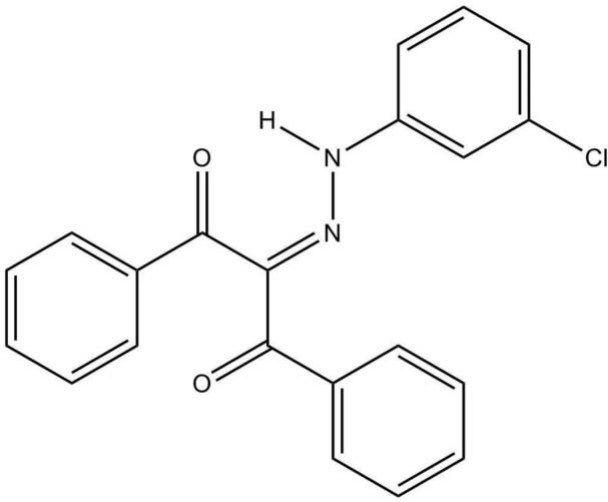


## Experimental

### 

#### Crystal data

C_21_H_15_ClN_2_O_2_
*M* *_r_* = 362.80
Monoclinic, 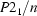

*a* = 10.2970 (9) Å
*b* = 10.3526 (9) Å
*c* = 16.8926 (15) Å
β = 102.131 (1)°
*V* = 1760.6 (3) Å^3^
*Z* = 4
Mo *K*α radiation
μ = 0.24 mm^−1^
*T* = 297 K
0.60 × 0.31 × 0.23 mm

#### Data collection

Bruker D8 Discover with SMART CCD area-detector diffractometer
11123 measured reflections
3573 independent reflections
2657 reflections with *I* > 2σ(*I*)
*R*_int_ = 0.042

#### Refinement

*R*[*F* ^2^ > 2σ(*F* ^2^)] = 0.042
*wR*(*F* ^2^) = 0.119
*S* = 0.98
3573 reflections
239 parameters
H atoms treated by a mixture of independent and constrained refinement
Δρ_max_ = 0.39 e Å^−3^
Δρ_min_ = −0.16 e Å^−3^

### 

Data collection: *SMART* (Bruker, 2001 ▶); cell refinement: *SAINT* (Bruker, 2000 ▶); data reduction: *SAINT*; program(s) used to solve structure: *SHELXS97* (Sheldrick, 2008 ▶); program(s) used to refine structure: *SHELXL97* (Sheldrick, 2008 ▶); molecular graphics: *XP* in *SHELXTL/PC* (Sheldrick, 2008 ▶); software used to prepare material for publication: *PLATON* (Spek, 2009 ▶) and *Mercury* (Macrae *et al.*, 2006 ▶).

## Supplementary Material

Crystal structure: contains datablocks global, I. DOI: 10.1107/S1600536811017909/pk2324sup1.cif

Structure factors: contains datablocks I. DOI: 10.1107/S1600536811017909/pk2324Isup2.hkl

Supplementary material file. DOI: 10.1107/S1600536811017909/pk2324Isup3.cml

Additional supplementary materials:  crystallographic information; 3D view; checkCIF report

## Figures and Tables

**Table 1:** Hydrogen-bond geometry (Å, °)

*D*—H⋯*A*	*D*—H	H⋯*A*	*D*⋯*A*	*D*—H⋯*A*
N1—H1⋯O2	0.922 (17)	1.930 (18)	2.6205 (18)	130.1 (14)
C12—H12⋯N2	0.93	2.55	3.016 (2)	111
N1—H1⋯O1^i^	0.922 (17)	2.628 (17)	3.344 (3)	135.0 (13)
C6—H6⋯O1^i^	0.93	2.63	3.390 (2)	140

Symmetry code: (i) 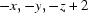.

**Fig. 1.**
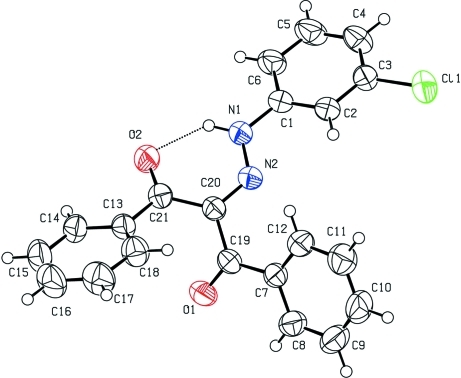
View of the title compound showing displacement ellipsoids for non-H atoms at the 50% probability level. The strong intramolecular hydrogen bond (N1-H1···O2) assisted by resonance (RAHB) is depicted by a dashed line.

**Fig. 2.**
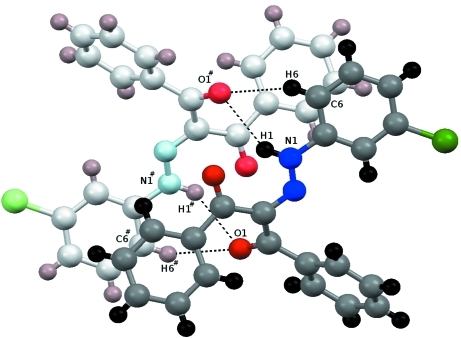
View of the formation of an inversion dimer via two weak intermolecular contacts (dashed lines). [Symmetry code: (#) -x, -y, 2 - z].

**Fig. 3.**
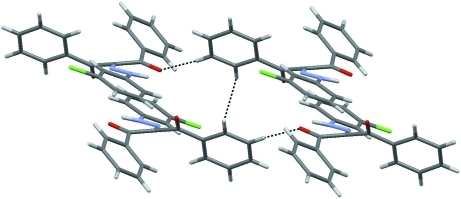
The dimers are connected via three weak contacts (dashed lines) to develop the three-dimensional structure.

## supplementary crystallographic information

### Comment

In recent years, much attention has been devoted to structural studies on heterodienic systems forming strong intramolecular hydrogen bonds, N—H···O, assisted by resonance (RAHB) which, *inter alia*, could have potential technological applications as bistate molecular switches (Olivieri *et al.*, 1989; Inabe, 1991; Bertolasi *et al.*, 1993; Bertolasi *et al.*, 1994*a*; Bertolasi *et al.*, 1994*b*; Krygowski *et al.*, 1997; Bustos *et al.*, 2007). On the other hand, it is well known that the phenyl diazonium salts are capable of coupling with a series of β-diketones that may yield the N—H···O moiety (Yao, 1964; Bustos *et al.*, 2007; Bustos *et al.*, 2009). In this report we present the crystal and molecular structure of the title compound 2-(2-(3-chlorophenyl)hydrazono)-1,3-diphenylpropane-1,3-dione, of formula 3-Cl—C_6_H_4_—NHN=C(COC_6_H_5_)_2_.

The molecular structure of the title compound exhibits one weak intramolecular hydrogen bond (C12–H12···N2) and one strong intramolecular hydrogen bond (N1–H1···O2) assisted by resonance (RAHB), see Fig. 1 and Table 2. The molecules form an inversion dimer *via* a pair of weak intermolecular contacts N1–H1···O1^#^ and C6–H6···O1^#^ hydrogen bonds [Symmetry code: (#) -*x*, -*y*, 2 - *z*], (see Fig. 2). These dimers are further stabilized *via* three weak contacts of the type shown in Fig. 3, to develop the three-dimensional structure.

### Experimental

Chemicals: 1,3-diphenylpropane-1,3-dione, 3-chloroaniline and sodium nitrite were procured from Sigma-Aldrich, and sodium hydroxide, sodium acetate, solvents and hydrochloric acid from Merck. Chemicals were used without further purification.

Procedure: In a 500 ml beaker were dissolved 2.24 g (0.01 mole) of 1,3-diphenylpropane-1,3-dione in 100 ml of an ethanol solution containing 0.4 g (0.01 mole) of sodium hydroxide and 3.65 g of sodium acetate.  The resulting β-diketonate solution was kept at -5°C and diluted with water to around 220 mL with vigorous stirring.  In another beaker, a diazonium ion solution was prepared adding 1.06 ml (0.01 mole) of 3-chloroaniline (99%, density 1.215 g/ml) in 8 ml of hydrochloric acid (5 mol/*L*), cooling at -5 °C, and adding a saturated aqueous solution containing 0.69 g (0.01 mole) of sodium nitrite. This solution was then added dropwise, with vigorous stirring, into the β-diketonate solution.  During the addition, a yellow solid precipitate was observed. This precipitate was filtered by suction and washed with an abundant quantity of water. Single crystals suitable for X-ray studies were obtained by recrystallization from a saturated solution of the compound in a 3:1 ethanol/acetone mixture.

### Refinement

All hydrogen atoms were found in difference Fourier maps.  The hydrogen attached to N1 was refined freely against the diffraction data, but all other H atoms were placed in geometrically idealized positions and constrained to ride on their parent atoms, with C—H = 0.93 Å and *U*_iso_(H) = 1.2*U*_eq_(C).

### Crystal data

**Table:**

C_21_H_15_ClN_2_O_2_	*F*(000) = 752
*M**_r_* = 362.80	*D*_x_ = 1.369 Mg m^−^^3^
Monoclinic, *P*2_1_/*n*	Mo *K*α radiation, λ = 0.71073 Å
Hall symbol: -P 2yn	Cell parameters from 999 reflections
*a* = 10.2970 (9) Å	θ = 2.3–26.3°
*b* = 10.3526 (9) Å	µ = 0.24 mm^−^^1^
*c* = 16.8926 (15) Å	*T* = 297 K
β = 102.131 (1)°	Polyhedron, yellow
*V* = 1760.6 (3) Å^3^	0.60 × 0.31 × 0.23 mm
*Z* = 4	

### Data collection

**Table:**

Bruker D8 Discover with SMART CCD area-detector diffractometer	2657 reflections with *I* > 2σ(*I*)
Radiation source: fine-focus sealed tube	*R*_int_ = 0.042
graphite	θ_max_ = 26.3°, θ_min_ = 2.3°
φ and ω scans	*h* = −12→12
11123 measured reflections	*k* = −12→12
3573 independent reflections	*l* = −20→20

### Refinement

**Table:**

Refinement on *F*^2^	Primary atom site location: structure-invariant direct methods
Least-squares matrix: full	Secondary atom site location: difference Fourier map
*R*[*F*^2^ > 2σ(*F*^2^)] = 0.042	Hydrogen site location: inferred from neighbouring sites
*wR*(*F*^2^) = 0.119	H atoms treated by a mixture of independent and constrained refinement
*S* = 0.98	*w* = 1/[σ^2^(*F*_o_^2^) + (0.071*P*)^2^] where *P* = (*F*_o_^2^ + 2*F*_c_^2^)/3
3573 reflections	(Δ/σ)_max_ = 0.001
239 parameters	Δρ_max_ = 0.39 e Å^−^^3^
0 restraints	Δρ_min_ = −0.16 e Å^−^^3^

### Special details

**Table:**

Geometry. Bond distances, angles *etc*. have been calculated using the rounded fractional coordinates. All su's are estimated from the variances of the (full) variance-covariance matrix. The cell e.s.d.'s are taken into account in the estimation of distances, angles and torsion angles
Refinement. Refinement on *F*^2^ for ALL reflections except those flagged by the user for potential systematic errors. Weighted *R*-factors *wR* and all goodnesses of fit *S* are based on *F*^2^, conventional *R*-factors *R* are based on *F*, with *F* set to zero for negative *F*^2^. The observed criterion of *F*^2^ > σ(*F*^2^) is used only for calculating -*R*-factor-obs *etc*. and is not relevant to the choice of reflections for refinement. *R*-factors based on *F*^2^ are statistically about twice as large as those based on *F*, and *R*-factors based on ALL data will be even larger.

### Fractional atomic coordinates and isotropic or equivalent isotropic displacement parameters (Å^2^)

**Table:**

	*x*	*y*	*z*	*U*_iso_*/*U*_eq_	
Cl1	0.69570 (5)	0.13603 (5)	1.18232 (3)	0.0776 (2)	
O1	−0.05070 (10)	0.25054 (10)	0.91373 (7)	0.0598 (4)	
O2	0.03738 (11)	−0.12050 (10)	0.92207 (7)	0.0630 (4)	
N1	0.25273 (13)	−0.04080 (12)	1.02183 (8)	0.0510 (4)	
N2	0.22035 (12)	0.07588 (11)	0.99272 (7)	0.0463 (4)	
C1	0.36632 (15)	−0.05568 (14)	1.08450 (9)	0.0459 (5)	
C2	0.46505 (14)	0.03750 (14)	1.09893 (9)	0.0477 (5)	
C3	0.57101 (15)	0.01951 (15)	1.16316 (9)	0.0516 (5)	
C4	0.58144 (17)	−0.08754 (18)	1.21159 (10)	0.0636 (6)	
C5	0.48391 (18)	−0.18002 (18)	1.19538 (11)	0.0696 (7)	
C6	0.37597 (17)	−0.16608 (16)	1.13176 (11)	0.0616 (6)	
C7	0.16409 (16)	0.33878 (14)	0.92641 (8)	0.0466 (5)	
C8	0.11665 (19)	0.46296 (15)	0.93282 (9)	0.0594 (6)	
C9	0.1988 (2)	0.56842 (17)	0.93377 (11)	0.0743 (8)	
C10	0.3278 (2)	0.55061 (18)	0.92704 (13)	0.0810 (8)	
C11	0.3759 (2)	0.42915 (18)	0.91881 (12)	0.0752 (7)	
C12	0.29480 (17)	0.32324 (16)	0.91906 (10)	0.0584 (6)	
C13	−0.05962 (15)	0.01258 (14)	0.81261 (9)	0.0489 (5)	
C14	−0.17413 (16)	−0.06169 (16)	0.79092 (10)	0.0580 (6)	
C15	−0.25909 (19)	−0.0430 (2)	0.71700 (12)	0.0768 (7)	
C16	−0.2295 (2)	0.0471 (2)	0.66386 (12)	0.0840 (8)	
C17	−0.1161 (2)	0.1191 (2)	0.68373 (11)	0.0787 (8)	
C18	−0.02983 (18)	0.10304 (17)	0.75838 (10)	0.0632 (6)	
C19	0.06905 (15)	0.23015 (14)	0.92398 (8)	0.0466 (5)	
C20	0.11471 (14)	0.09339 (14)	0.93512 (9)	0.0457 (5)	
C21	0.02976 (14)	−0.01183 (15)	0.89255 (9)	0.0482 (5)	
H1	0.1934 (18)	−0.1079 (15)	1.0080 (10)	0.062 (5)*	
H2	0.46010	0.11030	1.06620	0.0570*	
H4	0.65340	−0.09750	1.25480	0.0760*	
H5	0.49060	−0.25330	1.22780	0.0840*	
H6	0.31090	−0.22970	1.12080	0.0740*	
H8	0.02860	0.47520	0.93650	0.0710*	
H9	0.16670	0.65130	0.93900	0.0890*	
H10	0.38330	0.62170	0.92810	0.0970*	
H11	0.46310	0.41810	0.91310	0.0900*	
H12	0.32800	0.24070	0.91430	0.0700*	
H14	−0.19350	−0.12410	0.82630	0.0700*	
H15	−0.33650	−0.09150	0.70320	0.0920*	
H16	−0.28700	0.05920	0.61400	0.1010*	
H17	−0.09640	0.17930	0.64710	0.0940*	
H18	0.04710	0.15240	0.77180	0.0760*	

### Atomic displacement parameters (Å^2^)

**Table:**

	*U*^11^	*U*^22^	*U*^33^	*U*^12^	*U*^13^	*U*^23^
Cl1	0.0598 (3)	0.0744 (3)	0.0886 (4)	−0.0128 (2)	−0.0070 (3)	−0.0046 (2)
O1	0.0440 (7)	0.0589 (7)	0.0723 (7)	0.0061 (5)	0.0030 (5)	0.0073 (5)
O2	0.0609 (7)	0.0489 (7)	0.0717 (8)	−0.0080 (5)	−0.0030 (6)	0.0076 (6)
N1	0.0465 (7)	0.0428 (7)	0.0574 (8)	0.0001 (6)	−0.0032 (6)	0.0035 (6)
N2	0.0444 (7)	0.0431 (7)	0.0488 (7)	0.0025 (5)	0.0041 (6)	0.0035 (5)
C1	0.0420 (8)	0.0442 (8)	0.0497 (8)	0.0059 (6)	0.0057 (6)	0.0021 (6)
C2	0.0480 (9)	0.0454 (8)	0.0476 (8)	0.0038 (7)	0.0055 (7)	0.0041 (7)
C3	0.0466 (9)	0.0535 (9)	0.0522 (9)	0.0017 (7)	0.0048 (7)	−0.0048 (7)
C4	0.0539 (10)	0.0756 (12)	0.0559 (10)	0.0080 (9)	−0.0007 (8)	0.0142 (9)
C5	0.0577 (11)	0.0677 (11)	0.0798 (12)	0.0069 (9)	0.0060 (9)	0.0309 (10)
C6	0.0514 (9)	0.0528 (9)	0.0769 (12)	0.0008 (8)	0.0049 (8)	0.0174 (8)
C7	0.0525 (9)	0.0449 (8)	0.0385 (8)	0.0011 (7)	0.0005 (6)	0.0010 (6)
C8	0.0708 (11)	0.0498 (9)	0.0534 (9)	0.0075 (8)	0.0036 (8)	−0.0013 (7)
C9	0.1002 (17)	0.0449 (10)	0.0701 (12)	0.0008 (10)	0.0002 (11)	−0.0019 (8)
C10	0.0874 (16)	0.0552 (12)	0.0907 (15)	−0.0228 (10)	−0.0031 (12)	0.0091 (10)
C11	0.0633 (12)	0.0671 (12)	0.0928 (14)	−0.0129 (9)	0.0112 (10)	0.0116 (10)
C12	0.0560 (10)	0.0485 (9)	0.0690 (11)	−0.0021 (8)	0.0095 (8)	0.0032 (8)
C13	0.0444 (8)	0.0509 (9)	0.0495 (9)	0.0012 (7)	0.0059 (7)	−0.0072 (7)
C14	0.0531 (10)	0.0561 (10)	0.0618 (10)	−0.0040 (7)	0.0051 (8)	−0.0151 (8)
C15	0.0591 (11)	0.0872 (14)	0.0755 (13)	−0.0045 (10)	−0.0051 (10)	−0.0279 (12)
C16	0.0810 (15)	0.1068 (17)	0.0526 (11)	0.0086 (13)	−0.0125 (10)	−0.0128 (11)
C17	0.0879 (15)	0.0923 (15)	0.0525 (11)	0.0040 (12)	0.0071 (10)	0.0058 (10)
C18	0.0635 (11)	0.0705 (11)	0.0536 (10)	−0.0049 (9)	0.0079 (8)	0.0025 (8)
C19	0.0468 (9)	0.0509 (9)	0.0390 (8)	0.0024 (7)	0.0020 (6)	0.0035 (6)
C20	0.0424 (8)	0.0456 (8)	0.0464 (8)	−0.0002 (6)	0.0029 (6)	0.0033 (6)
C21	0.0429 (9)	0.0468 (9)	0.0540 (9)	−0.0015 (7)	0.0081 (7)	0.0001 (7)

### Geometric parameters (Å, °)

**Table:**

Cl1—C3	1.7418 (17)	C13—C21	1.487 (2)
O1—C19	1.2269 (19)	C14—C15	1.380 (3)
O2—C21	1.2264 (19)	C15—C16	1.373 (3)
N1—N2	1.3203 (17)	C16—C17	1.366 (3)
N1—C1	1.411 (2)	C17—C18	1.392 (3)
N2—C20	1.3107 (19)	C19—C20	1.491 (2)
N1—H1	0.922 (17)	C20—C21	1.485 (2)
C1—C6	1.386 (2)	C2—H2	0.9300
C1—C2	1.385 (2)	C4—H4	0.9300
C2—C3	1.380 (2)	C5—H5	0.9300
C3—C4	1.368 (2)	C6—H6	0.9300
C4—C5	1.373 (3)	C8—H8	0.9300
C5—C6	1.382 (3)	C9—H9	0.9300
C7—C8	1.388 (2)	C10—H10	0.9300
C7—C19	1.486 (2)	C11—H11	0.9300
C7—C12	1.387 (2)	C12—H12	0.9300
C8—C9	1.379 (3)	C14—H14	0.9300
C9—C10	1.369 (3)	C15—H15	0.9300
C10—C11	1.369 (3)	C16—H16	0.9300
C11—C12	1.379 (3)	C17—H17	0.9300
C13—C14	1.391 (2)	C18—H18	0.9300
C13—C18	1.389 (2)		
			
Cl1···C21^i^	3.5703 (16)	C21···Cl1^i^	3.5703 (16)
Cl1···H10^ii^	3.1300	C4···H12^i^	2.9600
O1···C13	2.9882 (18)	C5···H12^i^	3.0100
O1···C18	3.083 (2)	C8···H16^vii^	3.0200
O1···O2^iii^	3.0602 (16)	C8···H8^viii^	2.9800
O1···C15^iv^	3.386 (2)	C9···H4^ix^	2.9800
O1···C6^iii^	3.390 (2)	C10···H11^ii^	3.1000
O2···O1^iii^	3.0602 (16)	C14···H5^x^	2.9100
O2···N1	2.6205 (18)	C15···H6^x^	3.0300
O2···N2	2.8570 (16)	C19···H18	2.6600
O2···C19^iii^	3.2328 (18)	C20···H12	2.7600
O2···C20^iii^	3.1526 (19)	C20···H18	2.7700
O1···H15^iv^	2.6400	C21···H1	2.500 (17)
O1···H1^iii^	2.628 (17)	H1···O2	1.930 (18)
O1···H6^iii^	2.6300	H1···C21	2.500 (17)
O1···H8	2.4700	H1···H6	2.3900
O2···H1	1.930 (18)	H1···O1^iii^	2.628 (17)
O2···H9^v^	2.7000	H2···N2	2.5400
O2···H14	2.5800	H4···C9^vii^	2.9800
N1···O2	2.6205 (18)	H5···C14^xi^	2.9100
N2···O2	2.8570 (16)	H6···H1	2.3900
N2···C12	3.016 (2)	H6···O1^iii^	2.6300
N2···H2	2.5400	H6···C15^xi^	3.0300
N2···H12	2.5500	H8···O1	2.4700
C1···C14^iii^	3.402 (2)	H8···C8^viii^	2.9800
C6···C14^iii^	3.567 (2)	H8···H8^viii^	2.4000
C6···O1^iii^	3.390 (2)	H9···O2^xii^	2.7000
C10···C11^ii^	3.577 (3)	H10···Cl1^ii^	3.1300
C11···C10^ii^	3.577 (3)	H11···C10^ii^	3.1000
C12···N2	3.016 (2)	H12···N2	2.5500
C13···O1	2.9882 (18)	H12···C20	2.7600
C14···C1^iii^	3.402 (2)	H12···C4^i^	2.9600
C14···C6^iii^	3.567 (2)	H12···C5^i^	3.0100
C15···O1^vi^	3.386 (2)	H14···O2	2.5800
C18···C19	3.065 (2)	H15···O1^vi^	2.6400
C18···O1	3.083 (2)	H16···C8^ix^	3.0200
C19···O2^iii^	3.2328 (18)	H18···C19	2.6600
C19···C18	3.065 (2)	H18···C20	2.7700
C20···O2^iii^	3.1526 (19)		
			
N2—N1—C1	118.94 (12)	C19—C20—C21	119.83 (13)
N1—N2—C20	120.55 (12)	N2—C20—C19	114.55 (13)
N2—N1—H1	119.5 (11)	C13—C21—C20	120.28 (13)
C1—N1—H1	120.6 (11)	O2—C21—C13	119.97 (14)
N1—C1—C2	121.18 (13)	O2—C21—C20	119.69 (13)
N1—C1—C6	118.03 (14)	C1—C2—H2	121.00
C2—C1—C6	120.79 (15)	C3—C2—H2	121.00
C1—C2—C3	118.30 (14)	C3—C4—H4	121.00
C2—C3—C4	121.95 (15)	C5—C4—H4	121.00
Cl1—C3—C4	119.19 (12)	C4—C5—H5	119.00
Cl1—C3—C2	118.86 (12)	C6—C5—H5	119.00
C3—C4—C5	118.91 (16)	C1—C6—H6	121.00
C4—C5—C6	121.16 (17)	C5—C6—H6	121.00
C1—C6—C5	118.87 (16)	C7—C8—H8	120.00
C12—C7—C19	123.77 (14)	C9—C8—H8	120.00
C8—C7—C12	118.60 (15)	C8—C9—H9	120.00
C8—C7—C19	117.57 (15)	C10—C9—H9	120.00
C7—C8—C9	120.61 (18)	C9—C10—H10	120.00
C8—C9—C10	119.72 (17)	C11—C10—H10	120.00
C9—C10—C11	120.67 (18)	C10—C11—H11	120.00
C10—C11—C12	119.85 (19)	C12—C11—H11	120.00
C7—C12—C11	120.52 (16)	C7—C12—H12	120.00
C14—C13—C21	118.29 (14)	C11—C12—H12	120.00
C14—C13—C18	119.45 (15)	C13—C14—H14	120.00
C18—C13—C21	122.22 (14)	C15—C14—H14	120.00
C13—C14—C15	120.15 (16)	C14—C15—H15	120.00
C14—C15—C16	120.11 (18)	C16—C15—H15	120.00
C15—C16—C17	120.38 (19)	C15—C16—H16	120.00
C16—C17—C18	120.48 (18)	C17—C16—H16	120.00
C13—C18—C17	119.41 (17)	C16—C17—H17	120.00
O1—C19—C20	117.52 (13)	C18—C17—H17	120.00
O1—C19—C7	120.69 (13)	C13—C18—H18	120.00
C7—C19—C20	121.79 (13)	C17—C18—H18	120.00
N2—C20—C21	124.79 (13)		
			
C1—N1—N2—C20	−179.27 (14)	C8—C9—C10—C11	0.5 (3)
N2—N1—C1—C2	−20.0 (2)	C9—C10—C11—C12	−1.4 (3)
N2—N1—C1—C6	159.53 (14)	C10—C11—C12—C7	0.9 (3)
N1—N2—C20—C19	163.92 (13)	C18—C13—C14—C15	−1.8 (2)
N1—N2—C20—C21	−5.6 (2)	C21—C13—C18—C17	178.47 (16)
N1—C1—C6—C5	−177.52 (15)	C14—C13—C21—O2	30.3 (2)
C2—C1—C6—C5	2.0 (2)	C14—C13—C21—C20	−152.55 (15)
N1—C1—C2—C3	177.48 (14)	C18—C13—C21—O2	−147.25 (16)
C6—C1—C2—C3	−2.1 (2)	C18—C13—C21—C20	29.9 (2)
C1—C2—C3—Cl1	−179.79 (12)	C21—C13—C14—C15	−179.39 (16)
C1—C2—C3—C4	0.8 (2)	C14—C13—C18—C17	0.9 (3)
Cl1—C3—C4—C5	−178.97 (14)	C13—C14—C15—C16	1.4 (3)
C2—C3—C4—C5	0.4 (3)	C14—C15—C16—C17	−0.2 (3)
C3—C4—C5—C6	−0.5 (3)	C15—C16—C17—C18	−0.7 (3)
C4—C5—C6—C1	−0.8 (3)	C16—C17—C18—C13	0.3 (3)
C19—C7—C8—C9	−178.70 (14)	O1—C19—C20—N2	−136.93 (14)
C8—C7—C12—C11	0.5 (2)	C7—C19—C20—C21	−147.85 (13)
C8—C7—C19—O1	12.4 (2)	O1—C19—C20—C21	33.1 (2)
C8—C7—C19—C20	−166.55 (13)	C7—C19—C20—N2	42.09 (19)
C19—C7—C12—C11	177.53 (15)	N2—C20—C21—O2	16.7 (2)
C12—C7—C8—C9	−1.5 (2)	N2—C20—C21—C13	−160.48 (14)
C12—C7—C19—C20	16.4 (2)	C19—C20—C21—O2	−152.33 (14)
C12—C7—C19—O1	−164.61 (14)	C19—C20—C21—C13	30.5 (2)
C7—C8—C9—C10	1.0 (3)		

Symmetry codes: (i) −*x*+1, −*y*, −*z*+2; (ii) −*x*+1, −*y*+1, −*z*+2; (iii) −*x*, −*y*, −*z*+2; (iv) −*x*−1/2, *y*+1/2, −*z*+3/2; (v) *x*, *y*−1, *z*; (vi) −*x*−1/2, *y*−1/2, −*z*+3/2; (vii) *x*+1/2, −*y*+1/2, *z*+1/2; (viii) −*x*, −*y*+1, −*z*+2; (ix) *x*−1/2, −*y*+1/2, *z*−1/2; (x) *x*−1/2, −*y*−1/2, *z*−1/2; (xi) *x*+1/2, −*y*−1/2, *z*+1/2; (xii) *x*, *y*+1, *z*.

### Hydrogen-bond geometry (Å, °)

**Table:**

*D*—H···*A*	*D*—H	H···*A*	*D*···*A*	*D*—H···*A*
N1—H1···O2	0.922 (17)	1.930 (18)	2.6205 (18)	130.1 (14)
C12—H12···N2	0.93	2.55	3.016 (2)	111
N1—H1···O1^iii^	0.922 (17)	2.628 (17)	3.3435 (27)	135.0 (13)
C6—H6···O1^iii^	0.93	2.63	3.390 (2)	140

Symmetry codes: (iii) −*x*, −*y*, −*z*+2.
